# The natural history of Pelizaeus–Merzbacher disease caused by PLP1 duplication: A multiyear case series

**DOI:** 10.1002/ccr3.7814

**Published:** 2023-08-25

**Authors:** Angela M. Trepanier, Sienna Aguilar, John Kamholz, Jeremy J. Laukka

**Affiliations:** ^1^ Center for Molecular Medicine and Genetics Wayne State University School of Medicine Detroit Michigan USA; ^2^ Invitae, Inc. San Francisco California USA; ^3^ Department of Neurology University of Iowa Carver College of Medicine Iowa City Iowa USA; ^4^ Department of Medical Education University of Toledo College of Medicine & Life Sciences Toledo Ohio USA; ^5^ Department of Neurology University of Toledo College of Medicine & Life Sciences Toledo Ohio USA

**Keywords:** gene duplication, humans, mutation, myelin proteolipid protein, Pelizaeus–Merzbacher disease/genetics, Pelizaeus–Merzbacher disease/pathophysiology, phenotype

## Abstract

This study aimed to characterize the clinical features, developmental milestones, and the natural history of Pelizaeus–Merzbacher disease (PMD) associated with *PLP1* gene duplications. The study examined 16 PMD Patients ranging in age from 7 to 48 years, who had a documented *PLP1* gene duplication. The study examined and analyzed the medical and developmental histories of the subjects utilizing a combination of resources that included medical history questionnaires, medical record reviews, and a 31‐point functional disability scale that had been previously validated. The data extracted from the medical records and questionnaires for analysis included information related to medical and developmental histories, level of ambulation and cognition, and degree of functional disability. The summation of findings among the study population demonstrated that the presenting symptoms, developmental milestones achieved, and progression of symptoms reported are consistent with many previous studies of patients with *PLP1* duplications. All patients exhibited onset within the first year of life, with nystagmus predominating as the first symptom noticed. All patients exhibited delays in both motor and language development; however, many individuals were able to meet several developmental milestones. They exhibited some degree of continued motor impairment with none having the ability to walk independently. All patients were able to complete at least some of the cognition achievements and although not all were verbal, a number were able to use communication devices to complete these tasks. A critical tool of the study was the functional disability scale which provided a major advantage in helping quantify the clinical course of PMD, and for several, we were able to gather this information at more than one point in time. These reported findings in our cohort contribute important insight into the clinical heterogeneity and potential underlying mechanisms that define the molecular pathogenesis of the disease. This is one of only a small number of natural history studies examining the clinical course of a cohort of patients with *PLP1* duplications within the context of a validated functional disability scoring system. This study is unique in that it is limited to subjects with *PLP1* gene duplications. This study demonstrated many commonalities to other studies that have characterized the features of PMD and other PLP1‐related disorders but also provide significant new insights into the evolving story that marks the natural history.

## 1 INTRODUCTION

In 1885 Friedrich Pelizaeus, a German physician, first identified a genetic disorder in five boys in a single family with nystagmus, spasticity of the limbs, and developmental delay.^1^ Twenty‐five years later in 1910, Ludwig Merzbacher independently reexamined this family and described further the neuropathology of 14 affected individuals and found that all affected members shared a common ancestor.^2^ Together, Pelizaeus and Merzbacher identified this rare X‐linked inherited white matter disorder.

Pelizaeus–Merzbacher disease (PMD) is today recognized as part of a group of disorders caused by mutations in the *PLP1* gene. The gene encodes both PLP, a major component of central nervous system myelin, and an alternatively spliced isoform, DM20, which is a minor component of both central and peripheral nervous system myelin.^3^ Duplications of the *PLP1* gene cause the majority (50%–75%) of *PMD*. Point mutations in the coding or splice site regions are found in most of the remaining patients, although a very small portion is caused by deletions of the *PLP1* gene.^4^, ^5^ The disorder is thus genetically heterogeneous.

Individuals with *PLP1*‐related disorders are not only genetically heterogeneous but also clinically heterogeneous. They can, however, be loosely grouped into three main clinical phenotypes: (1) classic PMD, characterized by nystagmus, hypotonia, and delay in motor development with onset in the first year of life,^6^, ^7^ (2) connatal PMD characterized by severe hypotonia and stridor with onset at birth and death within the first 10 years of life,^6^, ^8^ and (3) SPG2, characterized by a slowly progressive X‐linked spastic paraparesis^3^, ^6^, ^8^ Classic PMD is usually caused by a duplication of the *PLP1* gene within its locus on the X‐chromosome and is typically described as slowly progressive; however, several patients have been observed to maintain a stable clinical course.^7^ Variations in the size of PLP1 duplications, and whether or not the region encompassed includes flanking genes, may contribute to variability. Connatal PMD is often caused by point mutations within the *PLP1* gene producing misfolding of PLP1, ER retention, and activation of the unfolded protein response (UPR). SPG2 is caused by PLP1 mutations that allow the protein to traverse the ER and become inserted in myelin. Other intermediate phenotypes depend on the specifics of the nature and location of the PLP1 mutation.

In order to facilitate future treatment interventions and to understand the natural history of PMD, in this work we have evaluated the clinical presentation and progression of a group of patients with PLP1 duplications. This was done both retrospectively, through chart review, and prospectively, by collecting clinical data. Taken together, these results will be useful for future treatment strategies, developing biomarkers, and timing of treatment interventions in this disease.

## 2 METHODS

### 2.1 Editorial policies and ethical considerations

This study was approved as an exempt study by Wayne State University Institution Review Board. Informed consent and assent, when applicable, were obtained from all participants.

### 2.2 Participants

Individuals, or the parents or guardians of individuals, who have a *PLP1*‐related disorder and an identified *PLP1* pathogenic variant, were invited to participate in the study. Participants were identified through one of three mechanisms. (1) Those who had previously participated in a *PLP1*‐related disorders (PLP1‐RD) study at Wayne State University/Detroit Medical Center (WSU/DMC) or who were participating in a different *PLP1* study at the time of enrollment; (2) those who had been seen/were being seen at WSU/DMC for clinical care or who had contacted the primary investigator because of a diagnosis of *PLP1‐RD*; (3) those who had genetic testing for *PLP1‐RD* through the molecular genetics laboratory at AI Dupont in Wilmington, DE. Recruitment was conducted either through mail or in person (for those who were being seen at WSU/DMC or Dupont during the period of enrollment). Potential participants/parents/guardians were sent or given a study packet that included the consent form with HIPAA authorization, an assent form (when applicable), a medical history questionnaire, a family history questionnaire, medical record release forms, and a “decline to participate” form. Individuals who had already filled out the questionnaires as part of ongoing/previous *PLP1*‐related disorders studies or clinical care instead received a follow‐up “current medical history” questionnaire. For all potential participants, those who did not return the “decline to participate” form within 2 weeks were contacted by telephone by a study investigator to answer questions about the study. Participants were also asked to consider taking part in an optional long‐term follow‐up which involves completing a one‐page “current medical history” questionnaire every 1–2 years.

### 2.3 Functional disability score

The functional disability score (FDS) is a clinical scale that has been developed and previously validated^9^ to analyze the clinical disability in patients with PLP1‐related disorders. The clinical scale measures the ability of patients to perform routine tasks of daily living. The scoring system does not depend on any one neurologic sign but is a reproducible scale that can collate responses from a patient's caregiver. The inter‐rater reliability of the scoring system is greater than 95% among a small team of neurologists at Wayne State University School of Medicine who estimated the functional disability of a group of 20 patients with genetically confirmed PMD.^9^ The FDS of this cohort of patients was determined by either direct examination of the patient, interview with the caregiver of the patient, and/or written report of the caregiver.

### 2.4 Analysis

Data were extracted from the medical records and questionnaires for analysis, including information related to medical and developmental histories, level of ambulation and cognition, and degree of functional disability. Medical and developmental histories, level of ambulation, and level of cognition were assessed based on questions from the medical history and current medical history questionnaires. The degree of functional disability was assessed based on the score from a 31‐point functional disability scale (FDS) from 0 (lowest level of achievement) to 31 (highest level of achievement).^9^ This score was derived from measures of nine areas of function: employment/education, speech, diet, dressing, toileting, drawing/writing, walking, sitting, and breathing (See Table 1). FDS scores were gathered from one of two mechanisms. (1) From chart review based on FDS scores previously obtained as part of prior participation in a PLP1‐RD study or as part of clinical care for PLP1‐RD or (2) from a series of questions asked in the medical history and current medical history questionnaires.

**TABLE 1:** Functional disability rating scale.^9^

Education	0—no formal schooling	1—special school or special education classes	2—regular classes, but not at grade level	3—regular school, grade‐appropriate for age (within 2 years)		
Employment^a^	0—unable to work/homebound	1—sheltered workshop	2—special job	3—regular job		
Speech	0—no verbal communication	1—rare understandable words with nonverbal communication	2—speech understandable with difficulty	3—detectable speech disturbance but readily understood	4—normal speech	
Feeding	0—tube feedings only	1—some oral feedings, with supplemental tube feedings	2—oral feedings with consistent changes in diet	3—normal diet with occasional choking	4—normal swallowing	
Dressing	0—total dependence	1—can assist with dressing but dependent on others	2—independent, but with decreased efficiency	3—normal		
Toileting	0—total dependence	1—needs assistance	2—independent, but with decreased efficiency	3—normal		
Writing	0—cannot reach for and grasp writing utensil	1—can reach for and grasp writing utensil, but cannot scribble	2—can scribble, but cannot draw or write letters	3—can draw or write letters	4—normal for age	
Sitting	0—cannot sit without support		2—can sit without support			
Walking	0—wheelchair or bedbound	1—can crawl/bunny hop	2—can walk a few steps, but needs adaptive aids or other support	3—needs adaptive aids to walk 20 feet	4—impaired gait, but uses no assistive devices	5—normal gait
Breathing	0—ventilator or constant respiratory support	1—intermittent use of non‐invasive respiratory support	2—has respiratory symptoms, but does not use ventilator support	3—normal breathing		

*Note*: Total score (out of 31 points).

^a^ Used if beyond school age (instead of education).

## 3 RESULTS

Sixteen subjects/parents or guardians completed the medical history questionnaire (MHQ) and medical record requests (See Table 2). The average age of the study subject at the time of completion was 22 years (range 7–48 years). Sixteen subjects had at least one functional disability scale score available. The average age at which the first functional disability scale (FDS1) score was obtained was 19 years (range 7–42 years; standard deviation 10.7 years). Nine of those individuals had at least two functional disability scale scores available. The average age at which the second functional disability scale (FDS2) score was obtained was 29 years (range from 14 to 48 years). The average number of years of follow‐up from FDS1 to FDS2 was 5.4 years (range 4–7 years). Two individuals had a third functional disability scale score available. The average age at which the third functional disability scale (FDS3) score was obtained was 48.5 years (range 46–51 years). The average number of years of follow‐up from FDS2 to FDS3 was 5.5 years. In total, three sibling pairs were included in this study.

**TABLE 2:** Demographic information.

Participant	Age MHQ1 (years)	Age FDS1 (years)	Age FDS2 (years)	F/U FDS1‐2 (years)	Age FDS3 (years)	F/U FDS2‐3	Sib pair (letter)
1	18	18	—	—	—	—	—
2	18	11	18	7	—	—	—
3	20	16	20	4	—	—	—
4	14	10	14	4	—	—	—
5	48	42	48	6	—	—	A
6	20	16	20	4	—	—	B
7	24	24	30	6	—	—	—
8	21	15	21	6	—	—	—
9	18	18	—	—	—	—	B
10	41	35	41	6	46	5	A
11	45	39	45	6	51	6	A
12	12	12	—	—	—	—	—
13	16	16	—	—	—	—	—
14	12	12	—	—	—	—	—
15	11	11	—	—	—	—	C
16	7	7	—	—	—	—	C
Average (SD)	21.6 (12.3)	18.9 (10.7)	28.6 (19.1)	5.4 (0.7)	48.5	5.5	—

Abbreviations: F/U, years follow‐up; FDS, functional disability scale; MHQ1, medical history questionnaire 1.

### 3.1 Medical and developmental histories in PMD

#### 3.1.1 Presenting symptoms

For a majority of the subjects in this study, the first symptom identified was nystagmus (11 of 16, 68.8%). In most cases, nystagmus was an isolated symptom but 3 of the 11 cases (27.3%) each had one additional symptom. These included seizures, “couldn't lift legs,” and head lag in a baby that also had cleft lip and palate. In the remaining subjects, the presenting symptoms included “missed developmental milestones,” “could not sit up,” “head lag,” “lower extremity tremors,” and “strabismus.” The average age at which the presenting symptom was noticed was 3.1 months (range from birth to 12 months; SD = 3.4 months).

#### 3.1.2 Common features/symptoms

Ninety‐four percent (15/16) of the subjects were reported to have had nystagmus at some point in their life. The average age at which nystagmus was noticed was 2.3 months (range from birth to 9 months, SD = 2.5, *n* = 12 subjects for whom age of onset was available).

All subjects reportedly had hypotonia (15 of 15). For most, the age of onset was between birth to 12 months (9/10 responses). For one individual, the onset of hypotonia occurred after a car accident when the individual was in his 30s.

Sixty‐three percent (10/16) of subjects had feeding problems. For the five subjects for which the age of onset was reported, it varied from less than 1 year of age to 31 years of age.

Forty percent (6 of 15) of subjects had gastroesophageal reflux. Age of onset was reported for five subjects and ranged from birth to 15 years of age (average age 6). All subjects reported to have gastroesophageal reflux were also reported to have feeding problems; however, not all subjects who had feeding problems also had reflux.

#### 3.1.3 Age at diagnosis

The average age of diagnosis was 5.1 years, ranging from birth to 18 years. This includes diagnoses made prenatally or at birth because of a previously affected sibling.

#### 3.1.4 Developmental milestones

Table 3 shows what developmental milestones subjects achieved and when available, the age at which they achieved them. Of those who responded to the developmental milestone questions, all (12 of 12) reported that the affected individual was able to hold their head up and turn back to the front (10 of 10). Ninety‐one percent (10 of 11) were able to turn front to back. Fifty‐four percent (6 of 11) were able to crawl either combat and/or belly style. Thirty‐six percent (4 of 11) were able to pull to a sit but only 17% (2 of 12) were able to sit alone. Thirty‐one percent (4 of 13) were able to take their first steps, one “with help,” one “with a kiddie walker,” and one “with assistance and support.” None (12 of 12) were able to stand alone, and none (11 of 11) were able to climb stairs. Thirty‐three percent (4 of 12) were able to pedal a tricycle. Regarding toileting, 60% (9 of 15) had achieved toilet training.

**TABLE 3:** Developmental milestones.

Patient	Held head up (months)	Turned back to front (months)	Turned front to back (months)	Crawled (months)	Sat alone (months)	Stood up alone	Took first steps (months)	Climb stairs	Babbles (months)	Spoke first words (months)	Spoke sentences (years)	Toilet trained (years)	Pedaled a tricycle (years)
1	5	4	4	12	−	−	−	−	10	12	3	3	−
2	5.5	7	8.5	−	−	−	−	−		−	−	−	−
3	WNR	Slight delay	Slight delay	WNR	−	−	−	−	WNR	12	4	5	−
4				7.5		−	−			12	3	−	−
5	7		9		24				3	9	2	6	
6	WNR	WNR	WNR	−	−	−	−	−	WNR	WNR	−	−	−
7				−	−	−	−	−		36		5	−
8	4	8	4				18		10	10		4	
9	3	6	6							12		8	
10	108								4	24			
11	10	8			18	−	−	−	6	12	2	2	−
12	12	+	+	−	−	−	−	−	7	−	−	+	−
13	18	+	+	+	−	−	+	−		+	−	−	+
14	4	8	8	24	−	−	120	−	60	60	−	−	6
15				36	−	−	24	−	3	17	2	11	3
16			−	−	−	−	−	−		−	−	−	6
Range in months	3–108	4–8	4–9	7–36	18–24		18–120		3–60	9–60	2–4	2–11	3–6

*Note*: Ages are in months. +, achieved, age not available; WNR+, achieved within the normal range for milestone; −, did not achieve; Blank, missing data.

In terms of language development, 83% (10 of 12) demonstrated the ability to babble, 81% (13 of 16) were able to speak their first words, and 50% (6 of 12) were able to speak in sentences.

#### 3.1.5 Ambulation

None of the study subjects (0 of 16) were able to walk unassisted; however, none were bedbound. Ninety‐four percent (15 of 16) reported that they currently use a wheelchair “all of the time” and the remaining individual reported using a wheelchair “most of the time.” Thirty‐eight percent (6 of 16) reported that they currently used braces “always,” and 6% (1 of 16) reported that they currently used a walker “most of the time.” Nineteen percent (3 of 16) reported using other devices (crutches, stander, and gait trainer). None of the participants (0 of 16) reported using a cane.

Seventy‐five percent (12 of 16) of subjects reported using a wheelchair starting at 0–10 years of age, 6% (1 of 16) starting at 10–20 years, and 6% (1 of 16) starting at 20–30 years. Sixty‐nine percent (11 of 16) reported using braces starting at 0–10 years of age; 25% (4 of 16) reported first using a walker at 0–10 years of age; and 6% (1 of 16) reported first using a walker at 10–20 years of age.

#### 3.1.6 Cognition

All subjects (15 of 15) were reported to know or respond to their names and were able to follow 2 step commands. Ninety‐three percent (14 of 15) could name two objects in the room, 86% (12 of 14) could add, and 77% (10 of 13) knew their address. At least some of the subjects required the use of communication devices to complete these tasks. Sixty‐nine percent (11 of 16) were reported to be able to read. Responses regarding reading level varied widely and ranged from “a little” or “letters” up to “12th‐grade” reading level.

### 3.2 Functional disability

#### 3.2.1 FDS overall scores

The average score for FDS1 was 11.5 (range 4–21; SD 5.1) (See Table 4). The average score of FDS2 was 11 (range 5–18.5; SD 9.5). The average score of FDS3 was 9 (range 4–14). The overall FDS1 scores were not significantly correlated with patient age (Pearson correlation −0.02‐tailed significance 0.907).

**TABLE 4:** Overall FDS scores.

Participant	FDS1	Change FDS1‐2	FDS2	Change FDS2‐3	FDS3
1	16	—	—	—	—
2	8	−3	5	—	—
3	13	0	13	—	—
4	19	−6.5	12.5	—	—
5	4	2	6	—	—
6	5	7.5	12.5	—	—
7	14	−2	12	—	—
8	15	−2.5	12.5	—	—
9	14			—	—
10	6	1	7	−3	4
11	21	−2.5	18.5	−4.5	14
12	7.5	—	—	—	—
13	9.5	—	—	—	—
14	10	—	—	—	—
15	15	—	—	—	—
16	6.5	—	—	—	—
Average	11.5 (5.1)	−0.7 (0.4)	11 (9.5)	−3.75	9

The average change in FDS scores from FDS1 to FDS2 was −0.7 (range −6.5 to 7.5). Five individuals scored lower on FDS2 (average change −3.3), three scored higher (average change 3.5) and one remained unchanged. The average change in FDS scores from FDS2 to FDS3 was −3.75 (both scored lower).

FDS individual scores: Answers from the following nine individual categories included in the overall FDS were also analyzed. Answers from FDS1 were most often included in this analysis. However, the range of responses selected for each question included responses from any available FDSs (FDS1 through FDS3).

Education/employment: Regarding education, 75% of subjects (9 of 12) attended a special school or had special education classes (Table 5). Responses ranged from “regular school grade‐appropriate for age (within 2 years)” to “special school or special education classes,” with no participants selecting “no formal schooling.” Concerning employment, the most often selected response was sheltered workshop (i.e., works at an institution dedicated to disabled employees) (50%, 2 of 4); however, responses ranged from a special job (i.e., works at a conventional workplace, but requires special supervision) to unable to work/homebound. From FDS1 to FSD2, five subjects' scores in education/employment increased, one decreased, and three did not change.

**TABLE 5:** Individual FDS scores by component.

Age at MHQ1	FDS1 Total	FDS2 Total	FDS3 Total	1‐Education	2‐Education	1‐Employment	2‐Employment	3‐Employment	1‐Speech	2‐Speech	3‐Speech	1‐Diet	2‐Diet	3‐Diet	1‐Dressing	2‐Dressing	3‐Dressing	1‐Toileting	2‐Toileting	3‐Toileting	1‐Drawing/Writing	2‐Drawing/Writing	3‐Drawing/Writing	1‐Walking	2‐Walking	3‐Walking	1‐Sitting	2‐Sitting	3‐Sitting	1‐Breathing	2‐Breathing	3‐Breatihing
18	16			1					2			4			1			3			2			0			0			3		
18	8	5		2	3				0	0		2	0		0	0		0	0		1	0		0	0		0	0		3	2	
20	13	13		1	1				2	2		3	4		1	0		1	1		2	2		0	0		0	0		3	3	
14	19	12.5		1	1				3	2.5		4	3		1	1		1	0		3	2		1	0		2	0		3	3	
48	4	6				0	1		0	0		0	0		0	0		0	0		2	3		0	0		0	0		2	2	
20	5	12.5		1	3				1	1		0	3		0	0		0	0.5		1	1		0	2		0	0		2	2	
24	14	12				2	0		2	2		4	3		1	1		1	1		1	2		0	0		0	0		3	3	
21	15	12.5		1	1				2	1.5		4	3		1	0.5		1	0.5		2	3		1	0		0	0		3	3	
18	14			2					2			3			0			1			2			1			0			3		
41	6	7	4			1	2	0	1	2	2	1	0	0	0	0	0	0	0	0	2	1	1	0	0	0	0	0	0	1	2	1
45	21	18.5	14			1	1.5	1	3	3	3	4	4	3	2	1	1	2	1	1	3	3	2	1	0	0	2	2	0	3	3	3
12	7.5			1					1			2			0.5			0			2			0			0			1		
16	9.5			1					1.5			3			0			0			2			0			0			2		
12	10			1					1			1			1			0			1.5			1.5			0			3		
11	15			3					2			3			0			0			2			2			0			3		
7	6.5			1					0			1			0			0			2			0.5			0			2		

Speech: The most often selected response was speech understandable, but with difficulty (37.5%, 6 of 16) with responses ranging from no verbal communication to detectable speech disturbance but easily understood. None of the participants selected normal speech. From FDS1 to FDS2, one participant's score for speech increased, three decreased, and six did not change.

Diet: The most often selected response was “normal swallowing” (5 of 16), with responses ranging from “normal swallowing” to “tube feedings only.” From FDS1 to FDS2, two participants' scores in diet increased, five decreased, and two did not change.

Dressing: The most often selected response was total dependence (50%, 8 of 16) with responses ranging from independent with decreased efficiency to total dependence. From FDS1 to FDS2, three participants' scores in dressing decreased and six did not change.

Toilet: The most often selected response was “total dependence” (9 of 16) with responses ranging from “normal” to “total dependence.” From FDS1 to FDS2, one participant's score for toileting increased, three decreased, and five did not change.

Drawing/writing: The most often selected response was can scribble but cannot draw or write letters (62.5%, 10 of 16), with responses ranging from can draw or write letters to cannot reach for and grasp writing utensil. None of the subjects were reported to be able to write/draw normally for their age. From FDS1 to FDS2, three participants' scores for drawing/writing increased, two decreased, and four did not change.

Walking: The most often selected response was “wheelchair or bedbound” (56%, 9 of 16), with responses ranging from can walk a few steps, but needs adaptive aids or other support to wheelchair or bedbound. From FDS1 to FDS2, one participant's score for walking increased, three decreased, and five did not change.

Sitting: The most often selected response was “cannot sit without support” (87.5%, 14 of 16). From FDS1 to FDS2, one participant's score for sitting decreased and the remaining eight did not change.

Breathing: The most often selected response was normal breathing (62.5%, 10 of 16), with responses ranging from normal breathing to intermittent use of non‐invasive respiratory support. None of the participants selected ventilator or constant respiratory support. From FDS1 to FDS2, one participant's score for breathing increased, one decreased, and seven did not change.

## 4 DISCUSSION

This study aimed to characterize the clinical features, developmental milestones, and the natural history of Pelizaeus–Merzbacher disease in a cohort of subjects, ranging in age from 7 to 48 years, who had a documented *PLP1* gene duplication (PMD). We examined and analyzed the medical and developmental histories of subjects utilizing medical history questionnaires, medical record reviews, and a 31‐point functional disability scale.^9^ Characterizing the natural history, to what extent the condition progresses over time, and the variability in both is important in providing genetic counseling and anticipatory guidance to parents and guardians of individuals with PMD. Understanding the natural history is also critical in the event that treatments to alter the disease course become available in the future.

The presenting symptoms, developmental milestones achieved, and progression of symptoms reported in our cohort were consistent with many previous studies of patients with *PLP1* duplications. All our patients exhibited onset within the first year of life, with nystagmus predominating as the first symptom noticed, consistent with previous reports.^7^, ^8^, ^10^, ^11^ In addition, most had nystagmus at some point in their lives and all had hypotonia, key characteristics of the classic PMD phenotype. Velasco Parra et al.^12^ reported on seven Columbian patients with Pelizaeus–Merzbacher disease ranging in age from 6 to 16 and with various *PLP1* pathogenic variants. Unlike in our cohort. In their series, only 28.7% (2 of 7) had early onset nystagmus and only 57% (4 of 7) had hypotonia. However, in a recent cohort study of 111 Chinese individuals with PMD and various PLP1 pathogenic variants who were followed for a median of 53 months, 99.1% (110/111) presented with nystagmus and 83.8% (93 of 111) with hypotonia.^13^

In our cohort, all of our subjects exhibited delays in both motor and language development; however, many individuals were able to meet several developmental milestones. Similar to previous studies, a subset of the PMD patients in our study were able to obtain head control, the ability to sit, the ability to speak several words or sentences, and some were even able to walk with assistance.^7^, ^8^, ^11^, ^13^ All individuals exhibited some degree of continued motor impairment with none of the participants having the ability to walk independently. We found that all individuals relied on the use of wheelchairs for most or all their ambulation. Like previous studies, the patients in our cohort seemed to exhibit large phenotypic variability.^11^ This variability occurred not only within the cohort but between siblings.

In terms of cognitive achievement, previous studies have observed that individuals with *PLP1* duplications often have some degree of intellectual disability, ranging from mild to severe.^7^, ^11^ All individuals in our cohort were able to complete at least some of the cognition achievements such as knowing or responding to their name and following two‐step commands. Although not all individuals were verbal, a number were able to use communication devices to complete these tasks. Additionally, many were able to read, although the reading levels were variable between individuals.

By utilizing the functional disability scale,^9^ we were able to quantify the clinical course of PMD, and for several individuals, we were able to gather this information at more than one point in time. The clinical course of PMD has previously been described as slowly progressive; however, to date, this has not been adequately characterized. In a study by Regis, et al.,^7^ five patients were followed for a period ranging from 5 to 12 years. In this study, the clinical course remained stable for four patients, while one showed a mild worsening in the last year of follow‐up. It is interesting to note that in our study population, there were individuals who scored both lower and higher on FDS2 versus FDS1 (as well as an individual with an FDS score that remained unchanged). Given the limited number of individuals in our study with more than one FDS score, a comparison between FDS1 and FDS2 scores was not significant; however, our study failed to depict a progressive clinical course. Given the limited number of follow‐up years in our study, it remains possible that our population is consistent with previous studies suggesting a slowly progressive disorder. Further research using a larger study population and FDS scores at additional time points would be necessary to characterize the clinical course more fully. Additionally, a pattern may exist whereby individuals with PMD gain skills for a period before deteriorating, as has been previously suggested^8^; however, our data set did not allow us to look for any such potential patterns.

## 5 CONCLUSION

This is one of only a small number of natural history studies examining the clinical course of a cohort of patients with *PLP1* pathogenic variants and is unique in that it is limited to subjects with *PLP1* gene duplications. This study demonstrated many commonalities with other studies that have characterized the features of PMD and other PLP1‐related disorders but also some new insights into the natural history.

There are several limitations of the current study. First, the size of the cohort is small (*n* = 16), with fewer individuals having completed a second or third FDS. This limitation is a reflection of the day‐to‐day demands that are required in providing time‐intensive care for PMD patients. For this reason, many statistical analyses were not possible. Given the small sample size, it may be difficult to generalize or extrapolate a conclusion from this study to a larger population of PMD patients, particularly given the extensive variability observed within and between families. Future research utilizing a larger cohort will be necessary to further clarify the natural history and clinical course of PMD. This work certainly provides a good foundation that opens a new window into the natural history of patients with *PLP1* duplications.

A second limitation arises due to the potential for inconsistency between measurements, given that some FDS scores were completed via self‐report, and others were completed based on in‐person physical examinations. Additionally, questionnaires were filled out based on the self‐report or report of a parent or guardian. An important strength is the ability to quantify and analyze the FDS through self‐report.

A third limitation was the small number of time points available for several of the participants, which limited our ability to comment on whether PMD was progressive. In addition, there were differences in the number of years of follow‐up between participants, and initial questionnaires and FDS scores were gathered at a wide range of ages.

Finally, there were several sibling pairs analyzed in the study. If the natural history and clinical course of PMD are assumed to vary less between members of the same family than it does between members of different families, the inclusion of multiple members of the same family in our study has the potential to bias our results.

## AUTHOR CONTRIBUTIONS

**Angela M. Trepanier:** Conceptualization; data curation; formal analysis; investigation; methodology; supervision; validation; writing – original draft; writing – review and editing. **Sienna Aguilar:** Data curation; formal analysis; methodology; writing – original draft. **John Kamholz:** Methodology; writing – review and editing. **Jeremy J. Laukka:** Writing – original draft; writing – review and editing.

## FUNDING INFORMATION

Some of the data used in this study was collected as part of a study funded by the European Leukodystrophy Association ELA (Grant # ELA 2011–02215).

## CONFLICT OF INTEREST STATEMENT

John Kamholz, Jeremy Laukka, and Angela Trepanier declare that there is no conflict of interest.

## CONSENT STATEMENT

Written informed consent was obtained from the patient to publish this report in accordance with the journal's patient consent policy.

## Data Availability

The data that support the findings of this study are available from the corresponding author upon reasonable request.
